# Daxx plays a novel role in T cell survival but is dispensable in Fas-induced apoptosis

**DOI:** 10.1371/journal.pone.0174011

**Published:** 2017-03-16

**Authors:** Jinghe Li, Liangyue Qian, John P. Dowling, Christine Curcione, Drishya Kurup, Jianke Zhang

**Affiliations:** 1 Department of Microbiology and Immunology, Sidney Kimmel Medical College, Thomas Jefferson University, Philadelphia, Pennsylvania, United States of America; 2 Sidney Kimmel Cancer Center, Thomas Jefferson University, Philadelphia, Pennsylvania, United States of America; Universite Paris-Sud, FRANCE

## Abstract

Daxx was originally isolated as a Fas-binding protein. However, the *in vivo* function of Daxx in Fas-induced apoptosis has remained enigmatic. Fas plays an important role in homeostasis in the immune system. Fas gene mutations lead to autoimmune-lymphoproliferation (*lpr*) diseases characterized by hyperplasia of secondary lymphoid organs. It is well established that the FADD adaptor binds to Fas, and recruits/activates caspase 8. However, additional proteins including Daxx have also been indicated to associate with Fas. It was proposed that Daxx mediates a parallel apoptotic pathway that is independent of FADD and caspase 8, but signals through ASK1-mediated apoptotic pathway. However, because the deletion of Daxx leads to embryonic lethality, the *in vivo* function of Daxx has not been properly analyzed. In the current study, analysis was performed using a conditional mutant mouse in which Daxx was deleted specifically in T cells. The data show that *Daxx*^*-/-*^ T cells were able to undergo normal Fas-induced apoptosis. While containing normal thymocyte populations, the T cell-specific *Daxx*^*-/-*^ mice have a reduced peripheral T cell pool. Importantly, Daxx-deficient T cells displayed increased death responses upon activation through TCR stimulation. These results unequivocally demonstrated that Daxx does not mediate Fas-induced apoptosis, but rather that it plays a critical role in survival responses in primary mature T cells.

## Introduction

Programmed cell death through apoptosis plays a critical role in the immune system. One of the major apoptotic signaling pathways is mediated by Fas (Apo-1 or CD95), a single transmembrane-domain protein of the neural growth factor receptor (NGFR)/tumor necrosis factor receptor (TNFR) super-family [1]. Basal level expression of Fas is detected in many tissues [2]. However, high levels of Fas are expressed in immature thymocytes and peripheral mature T cells. Fas is further induced upon activation of mature T cells [3,4].

Fas was originally identified as a cell surface antigen recognized by a monoclonal antibody which has a cytocidal effect on various tumor cells and primary cells [5,6]. The importance of Fas was first appreciated from the studies of a mutant mouse line which develops age-dependent lymphoproliferative (*lpr*) diseases manifesting lymphadenopathy and splenomegaly due to progressive accumulation of a peculiar T cell population which down-regulates the CD4 and CD8 co-receptors but expresses B220, a B cell-specific marker [7]. The *lpr* mice develop autoimmune diseases such as arthritis and symptoms similar to system lupus erythematosus (SLE), including high levels of autoantibodies. Studies mapped the Fas gene to the *lpr* locus and showed that the *lpr* mice carry mutant Fas alleles [8]. Importantly, targeted disruption of Fas led to *lpr* diseases, while transgenic expression of wild type Fas in lymphocytes corrected the phenotype in the naturally occurring Fas mutant mice [9,10].

The intracellular sequence of Fas contains a functional module called the death domain (DD) which is essential for apoptotic signaling [11,12]. When engaged to Fas ligand (FasL), Fas recruits the adaptor protein FADD (Fas-associated death domain or Mort1) through homotypic interactions between the two death domains [13–15]. FADD contains a second protein-protein interaction motif, the death effector domain (DED), which binds to the DED in pro-caspase 8 [16,17]. Once activated, the initiator caspase 8 cleaves and activates downstream effector caspases 3 and 7, leading to apoptosis [18,19].

In addition to FADD, other proteins such as Daxx were also identified as a potential Fas-interacting protein in yeast cells [20]. In mammalian cells, Daxx presumably binds to a region in the Fas death domain that is distinct from where FADD binds [20–22]. The interaction between the endogenous Fas-FADD is readily induced upon stimulation of Fas and can be detectable by conventional co-immunoprecipitation assays [13,23]. While overexpressed Daxx and Fas may be found in a complex, detecting the interaction of the endogenous Daxx and Fas has proved to be a challenge [20–22]. Daxx may also interact with cellular FLICE-like inhibitor protein long form (cFLIP_L_) [24], which is an enzyme-dead homologue of caspase 8 and plays an inhibitory role in Fas-induced apoptosis [25]. Furthermore, besides Fas signaling, Daxx may also be involved in TGF-β-induced apoptosis [26]. Unlike FADD, Daxx does not contain a DD or DED, and therefore it does not recruit caspase 8. Instead, it has been suggested that Daxx mediates Fas- and TGF-β-induced cell death through Ask1-Jnk activation [22,26]. However, some evidence also indicates that Fas activates Jnk independently of Daxx [26,27].

An earlier study of Daxx was performed by overexpressing a dominant-negative mutant, Daxx-DN, in cell lines, which concluded that Daxx was not involved in Fas-induced apoptosis [27]. However, a subsequent study indicated that Daxx-DN inhibited Fas-induced apoptosis in primary T cells [28]. Therefore, whether Daxx plays a role in the Fas-signaling pathway has remained an unresolved issue. Interestingly, the embryonic defects in Daxx knockout mouse did not appear to be due to lack of apoptosis as anticipated. Rather, massive apoptotic death was observed in *Daxx*^*-/-*^ embryos at day 8–9 of gestation [29]. In the current study, we employed conditional mutant mice in which Daxx was specifically deleted in T lineage cells in order to understand the physiological function of Daxx. Our data revealed a novel, temporal requirement for Daxx in peripheral mature T cells.

## Materials and methods

### Mice

Mice are housed in AAALAC-accredited vivarium at Thomas Jefferson University, and kept on autoclaved water and diet 5010 (LabDiet, St. Louis, MO) in ventilated racks. Nestlets were provided as enrichment. Cages get changed once every two weeks. The physical condition of mice was monitored at least once daily. A protocol is in place for the early euthanasia/humane endpoints for animals that became severely ill/moribund during the experiments. If mice were severely ill/moribund, displaying dramatic weight loss, rough hair/hunched back, and movement difficulties, euthanasia would be performed by CO_2_ inhalation. There was no illness or death prior to experimental endpoint. This study was carried out in strict accordance with the recommendations in the Guide for the Care and Use of Laboratory Animals of the National Institutes of Health. The protocol was approved by the Institutional Animal Care and Use Committee (IACUC) of the Thomas Jefferson University.

*LckCre* transgenic mice were described previously [30] and were originally obtained from Taconic and maintained on-site. Mice carrying the conditional knockout allele of *Daxx* (*Daxx*^*f/f*^) were created in Dr. P. Leder’s laboratory (Harvard Medical School). To generate T-cell specific Daxx-deficient mice, *Daxx*^*f/f*^ mice were crossed with *LckCre* transgenic mice. The resulting heterozygous *Daxx*^*+/f*^ *LckCre* mice were intercrossed to obtain T cell-specific Daxx-deficient, *Daxx*^*f/f*^ *LckCre* mice which were subsequently crossed with *Daxx*^*f/f*^ mice to produce *Daxx*^*f/f*^ *LckCre* (50%) mutant and *Daxx*^*f/f*^ (50%) control mice. To determine the genotypes, genomic DNA was extracted from the mouse tail, and used as templates for PCR amplification using allele-specific primers. Wild type and conditional knockout *Daxx* alleles were typed with primers 5’–AGCAGTAACTCCGGTAGTAGGAAG-3’ and 5’-AGGAACGGAACCACCTCAG-3’. The *LckCre* transgene was typed by PCR using primers 5’-CCGAAATTGCCAGGATCAGG-3’ and 5’-CTTACCTGTAGCCATTGCAGCTAG -3’. Mice are housed in an on-site facility and all procedures using mice were approved by the institutional animal care and use committee (IACUC) at Thomas Jefferson University.

### Western blotting analysis

Mice were euthanized and the indicated non-lymphoid organs or tissues were homogenized in RIPA lysis buffer containing 50 mM Tris-HCl, pH 7.4, 150 mM NaCl, 0.5% NP-40, 0.5% Na-deoxycholate, 0.1% SDS, 2 mM EDTA, 50 mM NaF, and a proteinase cocktail (Roche), followed by incubation on ice for 30 min. The spleen and lymph nodes were harvested, and single cell suspensions were prepared from these lymphoid organs. After red blood cell depletion by hypotonic lysis using ACK buffer, cells were enumerated using a Countess^®^ Automated Cell Counter (Invitrogen). Peripheral mature T cells were isolated by sorting for the Thy1.2^+^ population from total splenocytes and lymph node cells. These purified T cells were either left untreated or stimulated with plate-bound anti-CD3 Abs (1 μg/ml) plus soluble anti-CD28 Abs (1 μg/ml) for the indicated times. Resting or activated T cells were lysed in RIPA buffer or 1% Triton lysis buffer (50 mM Tris-HCl, pH 7.4, 150 mM NaCl, 1% Triton X-100, 20 mM EDTA and a proteinase cocktail) on ice for 30 min. Cell debris was removed by centrifugation at 14000 *g* for 5 min at 4°C. Total protein concentrations of the resulting lysates were determined using a Bio-Rad reagent (Cat#500–0006). Proteins (40 μg) were resolved by 10% or 15% PAGE, and western blotting performed using anti-Daxx and anti-p53 antibodies (Santa Cruz Biotechnology).

### Flow cytometric analysis of lymphocytes

For flow cytometric analysis, cells (~10^6^) from the indicated lymphoid organs were stained on ice for 30 min in 100 μl PBS containing 3% BSA, 1 mM EDTA, and 0.05% NaN_3_ with appropriate fluorochrome-conjugated antibodies against CD3, CD4, CD8, and B220. Data acquisition was performed using a Coulter Epics XL (Beckman Coulter) or a LSR II (Becton Dickinson) flow cytometer, and analyzed with the Flowjo software (Treestar). T cell numbers were estimated based on total cellularity and percentages of the CD3^+^ population in organs as indicated. Unpaired two-tailed *t*-tests were performed to obtain the *p* values, for comparison of total cellularity as well as total T cell numbers in mutant and control mice.

### T cell death assay

Total thymocytes were seeded into 96-well flat bottom plates (10^5^/well) in complete RPMI (10% FBS) and incubated at 37°C without additional stimulation for the indicated times, or with various concentrations of the Jo2 monoclonal anti-mouse Fas antibody (BD Pharmingen) plus cycloheximide (30 μg/ml; Sigma-Aldrich) for 14 h. Thymocytes were treated with the indicated concentrations of etoposide (Sigma-Aldrich) for 14 h at 37°C as described [31]. Mature T cells were isolated from the spleen and lymph nodes using the EasySep mouse T cell purification reagents (StemCell Technology), and stimulated with anti-CD3 ascites (1:1000 dilution) plus anti-CD28 ascites (1:1000 dilution) in round-bottom 96-well plates (10^5^/well). After incubating for 2 days at 37°C, these T cells were washed twice with PBS, and cultured in complete RPMI plus IL-2 (400 U/ml, NCI) for two days. The resulting activated T cells were treated with the indicated concentrations of soluble anti-Fas antibody or in wells coated with the indicated concentrations of anti-CD3 antibodies (2C11; BD Pharmingen) in flat-bottom 96-well plates (2 x 10^4^ /well). After incubation at 37°C for 16 h, T cells were stained with propidium iodide (PI, 1 μg/ml) and analyzed using a flow cytometer as described previously [32]. Death is indicated by percent of PI^+^ cells. Additionally, T cells were stained with annexin V-FITC and PI with a FITC Annexin V Apoptosis Detection Kit I (BD 556547), and two color flow cyometric analysis performed.

At the indicated times post stimulation with anti-CD3/CD28 ascites, cell cycle profiles were analyzed by determining DNA contents as previously described [33]. Briefly, cells were washed once with PBS containing 1% BSA and 0.01% NaN_3_, dispersed in 100 μl of a staining solution (0.1% sodium citrate, 0.1% Triton X-100, 50 μg/ml propidium iodide, 100 μg/ml RNaseA), and subsequently analyzed using a Coulter Epics XL or FACSCalibur flow cytometer.

### T cell proliferation assays

Mature T cells purified from the spleen and lymph nodes (10^5^) were seeded into 96-well round bottom plates in 100 μL of complete RPMI. After addition of anti-CD3 ascites at various dilutions and anti-CD28 ascites (1:1000 dilution), cells were incubated for 40 h at 37°C, followed by an 8 h pulse with 0.5 μCi of [^3^H] thymidine. Cells were collected using a 96-well harvester, and thymidine incorporation was determined by using a liquid scintillation counter. T cell accumulations were determined by enumeration using a hemocytometer, and proliferation was shown as bar graphs or growth curves. T cell division kinetics were analyzed using the CellTrace^™^ Violet Cell Proliferation Kit (Invitrogen), as per the manufacturer’s instruction. Briefly, 2μl of Cell Trace Violet (5 mM) in DMSO was added to 2 ml of purified T cells (10^6^/ml) in 2 ml PBS. After 20 min incubation at 37°C, the reaction was quenched by adding 10 ml complete RPMI 1640, followed by 5 min incubation at 37°C. After centrifugation, the labeled T cells were resuspended in 1 ml complete RPMI, and were seeded into wells (2×10^5^ cells/well) of 96-well round-bottom plates. Stimulation of T cells was initiated by adding anti-CD3 and anti-CD28 ascites (1:1000 dilution). Alternatively, 96-well flat-bottom plates were coated with anti-CD3 antibodies (1 μg/ml; BD Pharmingen 553058) for 2 h, and the labeled T cells were then seeded to each well. Anti-CD28 was added into each well at the concentration of 1 μg/ml (BD Pharmingen 553294). After incubation at 37°C for the indicated time, T cells were analyzed using a LSR II flow cytometer. Dead cells were detected by staining with propidium iodide (PI, 1 μg/ml).

### Caspase activity measurement

Caspase activity was determined using CellEvent Caspase-3/7 Green Flow Cytometry Assay Kit (Invitrogen), as per the manufacturer’s instruction. Mature T cells purified from the spleen and lymph nodes (10^5^) were seeded in 100 μL of complete RPMI into 96-well round bottom plates coated with anti-CD3 abs (1 μg/ml; BD Pharmingen 553058) for 2 h. Anti-CD28 abs (0.2 μg/ml; BD BioSciences 553294) were added into each well. After incubation at 37°C for the indicated time, CellEvent Caspase-3/7 Green Detection Reagent (400 nM) was then added into T cell suspensions for 30 min at 37°C and analyzed using an LSR II flow cytometer.

### T cell adoptive transfer

B6.CD45.1^+^ mice were obtained from The Jackson Laboratory and subjected to a 500rad irradiation. *Daxx*^*+/+*^ and *Daxx*^*-/-*^ donor T cells (CD45.2^+^) were isolated from the spleen and lymph nodes of indicated mice. T cells were resuspended in PBS and adoptively transferred into irradiated CD45.1^+^ recipient mice (3 x 10^6^ cells/mouse) by retroorbital injection. Seven days later, spleen and lymph nodes of recipient mice were made into single cell suspensions, stained with anti-CD45.1^+^ and CD45.2^+^ (eBioscience) and anti-Thy1.2 (BD Biosciences) antibodies, and analyzed by flow cytometry on an LSR II.

### Statistical analysis

Data are expressed using mean ± standard error of the mean (SEM). Comparison of results was performed using Student’s *t*-test. Statistical significance was defined as *p*<0.05 (*), *p*<0.01 (**), and *p*<0.001 (***).

## Results

### Tissue expression and conditional deletion of Daxx in T cells

**Fig 1:**
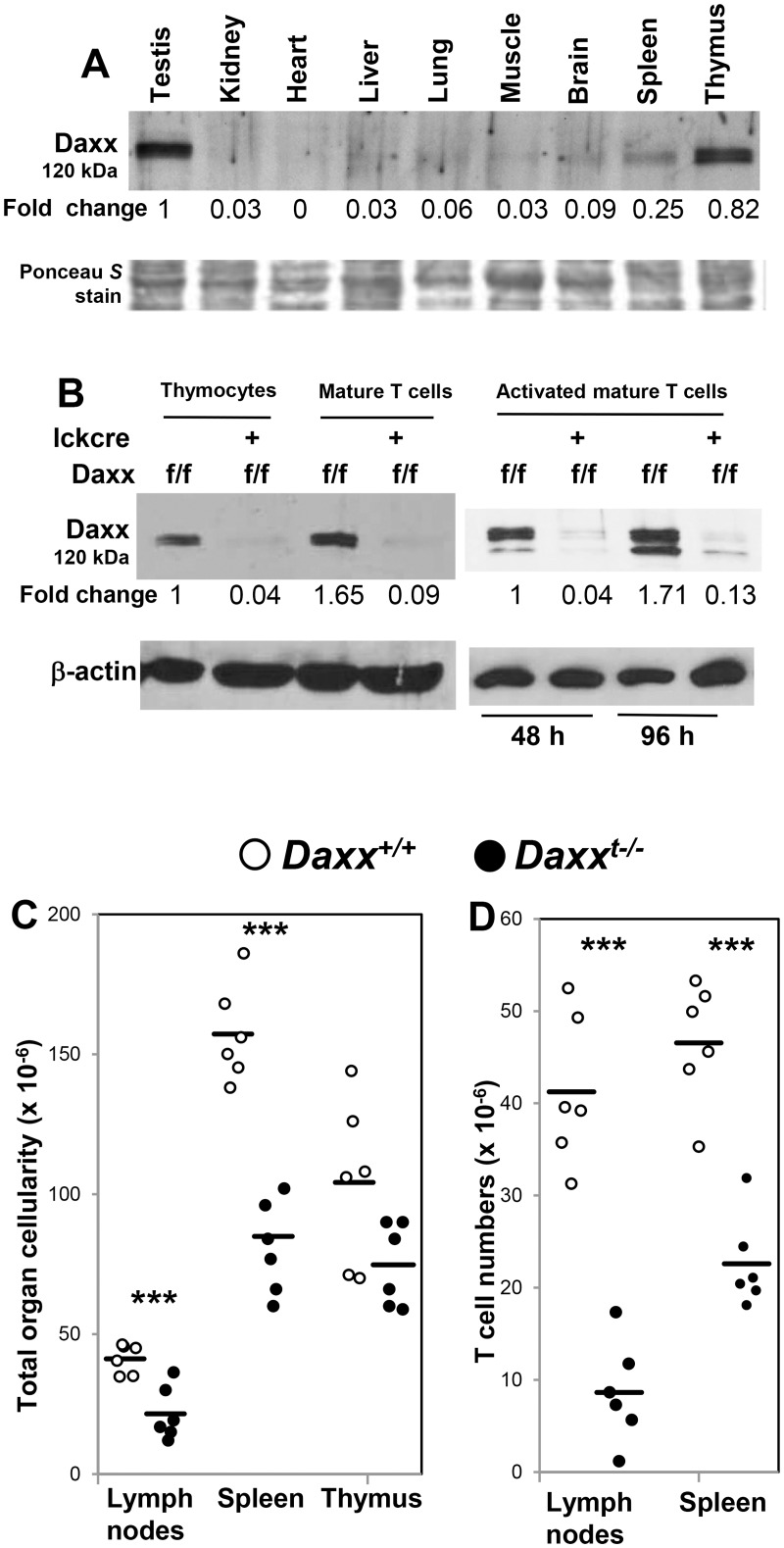
Tissue distribution and T cell-specific deletion of the Daxx protein. (A) The Daxx protein levels in the indicated organs of wild type adult mice were analyzed by western blotting. (B) To detect Daxx expression in T cells, total thymocytes or mature T cells sorted from the spleen and lymph nodes were prepared. Mature T cells were stimulated with anti-CD3 and CD28 antibodies for the indicated times (Activated T). Western blotting analysis was performed using antibodies against mouse Daxx. Fold changes were indicated below each blot. Ponceau *S* staining was performed as protein loading and transfer control (A). β-actin was used as blotting control (B). Total cell numbers in the indicated lymphoid organs (C), and T cell numbers (D) from the indicated organs of mutant mice (filled circles) and control mice (open circles) were shown. *** indicates *p* values < 0.05.

Since the initial cloning of Daxx as a potential Fas-interacting protein [20], its function in Fas-induced apoptosis has remained enigmatic [27,29]. Deletion of Daxx in germ cells leads to early embryonic lethality [29], which has prevented further analysis of the function of Daxx in the adult immune system. We performed western blot analysis to determine the expression of the Daxx protein in various tissues. Interestingly, we found that Daxx is not widely expressed in adult mice (Fig 1A). High levels of the Daxx protein were detected in the testis and thymus, and lower levels were detected in the spleen. Other tissues tested contain minimal levels of Daxx (Fig 1A). We performed a further analysis, using western blotting with mature T cells purified from the peripheral lymphoid organs. As shown in Fig 1B (Left panel), the Daxx protein is highly expressed in peripheral mature T cells, similar to that in thymocytes.

The apparently restricted tissue expression of Daxx prompted us to examine its physiological function in primary T cells, where Daxx is highly expressed (Fig 1A and 1B). Given the embryonic lethality in *Daxx*^*-/-*^ mice shown in the previous study [29], we performed lineage-specific deletion of Daxx using a conditional knockout mice (*Daxx*^*f/f*^), in which exon 2 of *Daxx* is flanked with two *loxP* sites. To induce T cell-specific deletion of Daxx, the *Daxx*^*f/f*^ mice were crossed with mice containing the T cell-specific *LckCre* transgene [30]. The expression of LckCre is initiated in pro/pre T cells as previously described [30,32]. Western blotting was performed to detect Daxx in the resulting mutant *Daxx*^*f/f*^ *LckCre* mice, using *Daxx*^*f/f*^ litter mates as a control which expresses Daxx at levels similar to *Daxx*^*+/+*^ wild type mice (data not shown). Deletion of the *Daxx* gene was evident, since the levels of the Daxx protein were dramatically reduced in thymocytes and purified mature T cells from *Daxx*^*f/f*^ *LckCre* mice compared to control *Daxx*^*f/f*^ mice (Left panel, Fig 1B). Peripheral mature T cells isolated from the spleen and lymph nodes were stimulated with anti-CD3 and anti-CD28 antibodies, and the resulting activated T cells from mutant *Daxx*^*f/f*^ *LckCre* mice contained minimal levels of the Daxx protein compared to T cells from control *Daxx*^*f/f*^ mice (Right panel, Fig 1B). Therefore, LckCre induced efficient deletion of Daxx in T cells. Hereafter, *Daxx*^*f/f*^ *LckCre* mice are also referred to as *Daxx*^*t-/-*^ mice.

### Daxx deletion does not lead to progressive accumulation of peripheral T cells, unlike the *lpr* symptom seen in Fas-deficient mice

**Fig 2:**
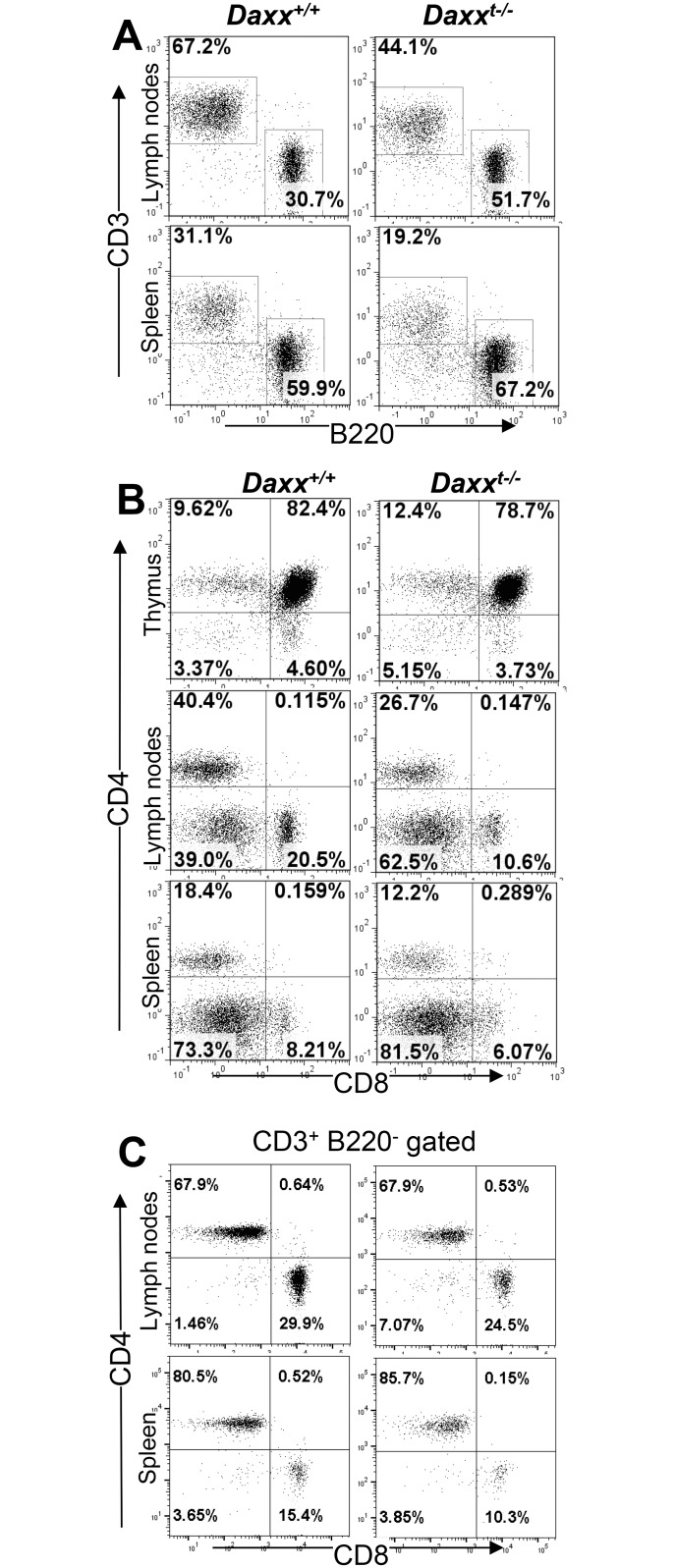
Analysis of T lymphocyte populations in *Daxx*^*t-/-*^ mice. Cells were isolated from the thymus, spleen, and lymph nodes in mutant *Daxx*^*t-/-*^ and *Daxx*^*+/+*^ control mice, stained for CD3 and B220 (A), or CD4, CD8, CD3, and B220 (B and C) and flow cytometric analysis was performed. Percentages represent the fraction of each indicated subpopulation of the lymphocytes gate. CD4 and CD8 expression was analyzed by gating on the CD3^+^B220^-^ population (C). Data are representative of at least 6 independent experiments.

To evaluate the impact of T cell-specific Daxx deficiency in the lymphoid compartment, the thymus, spleen, and lymph nodes were isolated from *Daxx*^*t-/-*^ mutants and total cell numbers in these organs were determined. As shown in Fig 1C, there was no significant difference in the thymic cellularity between *Daxx*^*t-/-*^ mutant and control mice. However, the total cell numbers in the lymph nodes and spleen appeared to be reduced significantly in *Daxx*^*t-/-*^ mutant mice, as compared to control mice (Fig 1C). Flow cytometry was performed to analyze the T lineage through staining for the T cell marker CD3, and the B cell marker, B220. CD3^+^ T cells were present in *Daxx*^*t-/-*^ mutant mice, although the percentages in the spleen and lymph nodes were lower compared to control mice (Fig 2A). The absolute T cell numbers in the lymph nodes and spleen of *Daxx*^*t-/-*^ mutant mice were lower than that of control mice (Fig 1D). Further flow cytometric analysis of T cell subsets was performed, revealing that the thymic T cell profile of *Daxx*^*t-/-*^ mutant mice, including the proportions of pro/pre T cells (CD4^-^CD8^-^), immature T cells (CD4^+^CD8^+^), and mature T cells (CD4^+^ or CD8^+^), is similar to that found in control *Daxx*^*+/+*^ mice (Fig 2B, top panel). However, the percentages of both the CD4^+^ and CD8^+^ T cell lineages in the peripheral lymphoid organs, including the spleen and lymph nodes, were lower in *Daxx*^*t-/-*^ mutant mice than in control mice (Fig 2B). This finding agrees with the observation showing a decrease in the total peripheral T cell numbers in mutant mice compared to control mice (Fig 1D). The CD4 and CD8 T cell profiles were analyzed by gating on the CD3^+^B220^-^ population, and the data indicated no effect on CD4 or CD8 expression in T cells (Fig 2C). There was no phenotypic difference among *Daxx*^*+/+*^, *Daxx*^*f/f*^, and *Daxx*^*+/+*^ *LckCre* control mice. Therefore, they were used as the wild type control hereafter and the *Daxx*^*+/+*^ controls include mice of all three genotypes.

### Normal Fas-induced apoptotic responses in Daxx-deficient T cells

**Fig 3:**
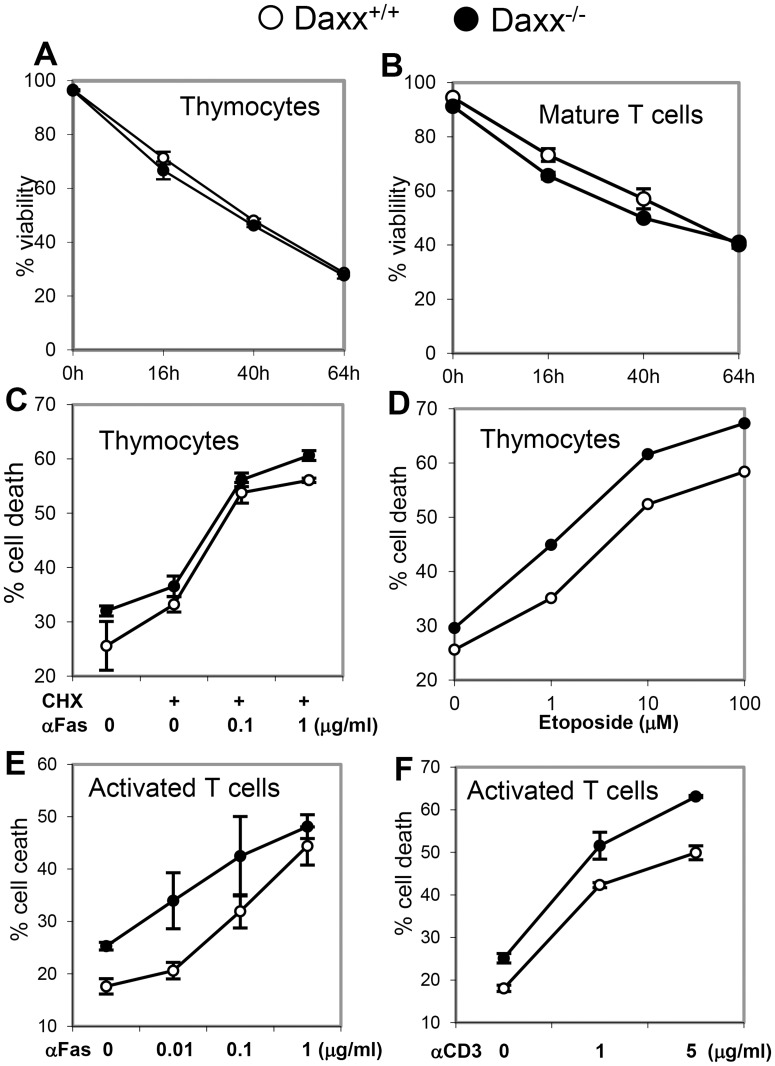
Survival and apoptosis in Daxx-deficient T cells. Thymocytes (A) or purified mature T cells (B) were cultured in complete medium without additional stimulation and viability was determined at the indicated times after incubation at 37°C. Thymocytes were treated with the indicated concentrations of anti-Fas antibodies (C) or etoposide (D) for 14 h. Mature T cells were stimulated with anti-CD3/CD28 antibodies and the resulting activated T cells were treated with anti-Fas (E) or anti-CD3 (F) antibodies for 16 h. T cells were stained with PI, and percentages (mean ± SEM of triplicates) of cell death (PI^+^) were determined using a flow cytometer. Data represents 3 independent experiments.

While Fas is widely expressed, its absence leads to pathology mostly restricted to the lymphoid system, without affecting mouse embryonic or postnatal development. Interestingly, although Daxx was originally identified as a possible mediator of Fas-induced apoptosis [20], *Daxx*^*-/-*^ mice die at an early embryonic stage due to massive death in various tissues, as shown previously [29]. To determine the effect of Daxx deficiency on death responses, T cells were isolated from the thymus, spleen or lymph nodes of *Daxx*^*t-/-*^ mutant and *Daxx*^*+/+*^ control mice and cultured in medium without additional stimulation. The percentages of viable cells at various times in *Daxx*^*-/-*^ thymocytes or peripheral mature T cell cultures were comparable to those in control *Daxx*^*+/+*^ T cells (Fig 3A and 3B). Therefore, deletion of Daxx does not alter *ex vivo* survival of T cells. We then analyzed T cell responses to Fas stimulation. Wild type thymic immature T cells readily undergo apoptosis when stimulated through Fas, and this response did not appear to be affected by Daxx deficiency (Fig 3C). Intrinsic death responses in *Daxx*^*-/-*^ T cells induced through treatment with etoposide were not impacted either (Fig 3D). Repeated stimulation through the TCR lead to apoptotic death that is mediated by Fas, a process referred to as activation-induced cell death (AICD) [34–36]. To determine whether Daxx plays a role in AICD, T cells were activated through challenges with anti-CD3 antibodies to crosslink the TCR, and then cultured in the presence of IL-2. The resulting activated T cells were treated with soluble anti-Fas antibodies or plate-bound anti-CD3 antibodies, and cell death was determined. We found that *Daxx*^*-/-*^ mutant and *Daxx*^*+/+*^ control T cells were both sensitive to killing by stimulation with anti-Fas antibodies or re-stimulation of the TCR with anti-CD3 antibodies (Fig 3E and 3F). Taken together, these results indicate that Daxx does not play a role in Fas-induced apoptosis or TCR activation-induced cell death (AICD) in T cells.

### Proliferative and death responses in activated *Daxx*^*-/-*^ T cells

**Fig 4:**
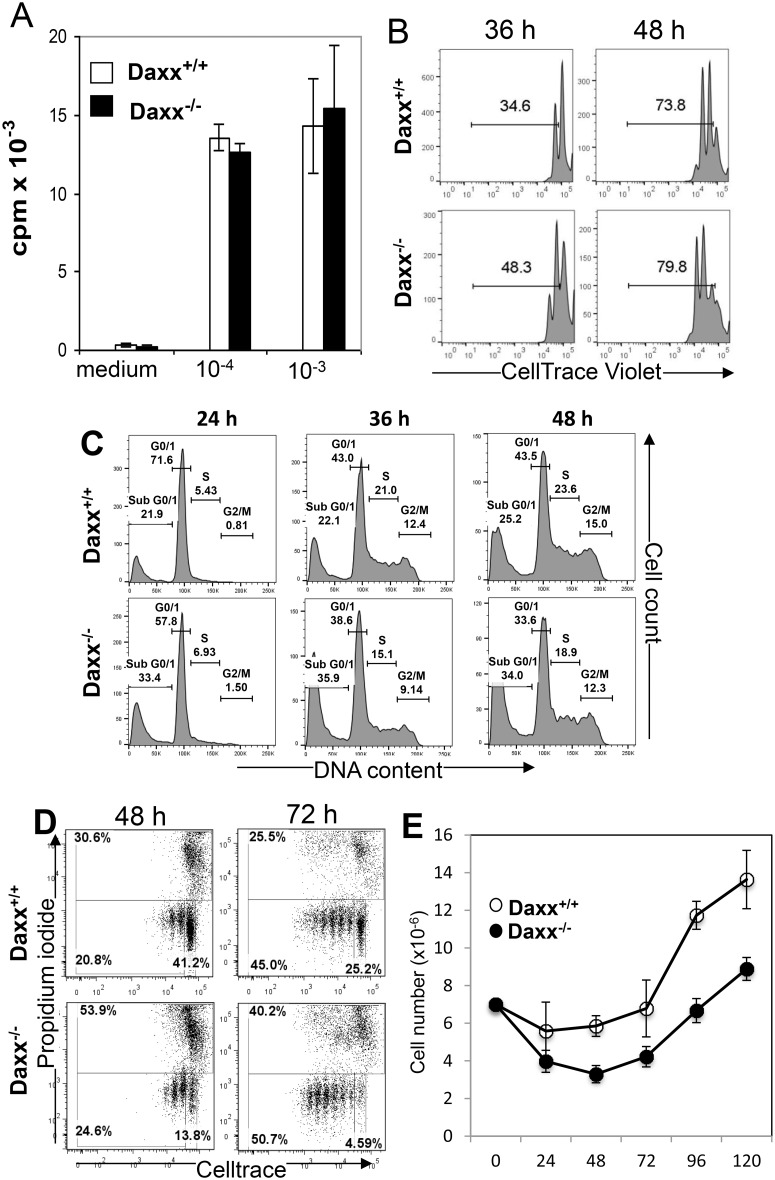
Analysis of cell proliferation and cell death responses of activated *Daxx*^*-/-*^ T cells. (A), Mature T cells purified from the spleen and lymph nodes in mice of the indicated genotypes were stimulated with anti-CD3/CD28 antibodies at the indicated dilutions (x-axis) for 48 h and proliferation was determined based on the amounts of tritiated thymidine (cpm) incorporated into activated T cells. Error bars indicate SEM of triplicates of one experiment, representing at least three independent experiments. (B), Mature T cells were labeled with CellTrace Violet^™^, stimulated with anti-CD3/CD28 antibodies for the indicated times, and analyzed using a flow cytometer. Percentages of divided cells are shown. Data represents 3 independent experiments. (C), Mature T cells were purified, stimulated with anti-CD3/CD28 antibodies as indicated in (A-B). DNA content was determined by PI staining and flow cytometry. Numbers indicate the percentages of cells in each cell cycle stage. (D), Mature T cells were labeled with CellTrace violet and activated as in (B) for the indicated times, stained with PI and analyzed by two-color flow cytometry. Percentages of undivided (CellTrace Violet^+^PI^-^) and divided cells (CellTrace Violet^lo^PI^-^) and cell death (PI^+^) are indicated. (E), Cell numbers at the indicated times following T cell activation were enumerated and growth curves plotted (mean ±SEM; n = 3).

To analyze the effect of Daxx deletion on T cell proliferative responses, spleen and lymph node cells were isolated from *Daxx*^*t-/-*^ and control *Daxx*^*+/+*^ littermates. T cells were purified and stimulated through the TCR and the co-stimulatory receptor CD28 with the corresponding agonistic antibodies. Relative T cell proliferation was determined by the amount of incorporation of [^3^H] thymidine into the DNA of T cells. *Daxx*^*-/-*^ mutant and *Daxx*^*+/+*^ control T cells appeared to proliferate similarly at 48 h post stimulation (Fig 4A). To further analyze T cell proliferation responses, we performed analyzed cell division kinetics by labeling purified T cells with the CellTrace Violet^™^ fluorescent dye and then stimulating them with agonistic anti-CD3 and anti-CD28 Abs. The dye is diluted as T cells divide, and the T cells undergoing cell divisions can be detected as distinct populations of decreasing intensities of CellTrace Violet fluorescence using a flow cytometer. As shown in Fig 4B, upon activation through TCR stimulation, divided cell populations were detected in *Daxx*^*-/-*^ mutant T cells (48.3% at 36 h and 79.8% at 48 h) and in *Daxx*^*+/+*^ T cells (34.6% at 36 h and 73.8% at 48 h). These results indicate that when Daxx is absent, T cells are able to undergo cell division induced by TCR signaling. It appears that mutant T cells divide more readily than control T cells (see also below).

In further analysis, we determined whether Daxx deficiency affected cell cycle progression. Purified T cells were stimulated with anti-CD3 and anti-CD28 antibodies, and the DNA content of activated T cells was analyzed by staining with propidium iodide. The percentage of cells at various cell cycle stages was determined by measuring the relative DNA content in the cells, as indicated by PI fluorescence using a flow cytometer. The percentages of cells in the *G*_*0/1*_, *S* and *G*_*2*_*/M* phase tend to be lower in mutant *Daxx*^*-/-*^ T cells than in control *Daxx*^*+/+*^ T cells (Fig 4C). Importantly, mutant *Daxx*^*-/-*^ T cells consistently had higher percentages of cells in the *sub-G*_*0/1*_ phase than the control *Daxx*^*+/+*^ T cells (Fig 4C). The *sub-G*_*0/1*_ phase population typically represents dying cells.

To analyze cell death responses versus proliferative responses further, we performed two color flow cytometry analysis. We used the CellTrace dye to label T cells and then activated them with anti-CD3 and -CD28 antibodies. Cell death was measured using propidium iodide (PI) uptake. There were fewer undivided cells in the *Daxx*^*-/-*^mutant (13.8%) than in the control (41.2%) at 48 h post stimulation (Fig 4D). Concomitantly, *Daxx*^*-/-*^ mutant T cells had more divisions than in *Daxx*^*+/+*^ control T cells (24.6% *vs*. 20.8%). These differences became more apparent at 72 h post TCR stimulation, where 4.6% of mutant T cells were undivided, a percentage much lower than the 25.2% of undivided control T cells. At this later time point, more division occurred in the mutant than in the control T cells (50.7% vs. 45.5%). Importantly, more cell death (53.9% at 48 h and 40.2% at 72 h) was observed in mutant than in control T cells (30.6% at 48 h and 25.5% at 72 h, Fig 4D). Finally, the numbers of activated T cells were determined and growth curves were generated, which indicate lower accumulation of *Daxx*^*-/-*^ mutant T cells than *Daxx*^*+/+*^ control T cells (Fig 4E). In total, a lack of Daxx consistently results in enhanced death responses in T cells (Fig 4C, 4D and 4E).

### Lack of Daxx leads to enhanced apoptosis in *Daxx*^*-/-*^ T cells

**Fig 5:**
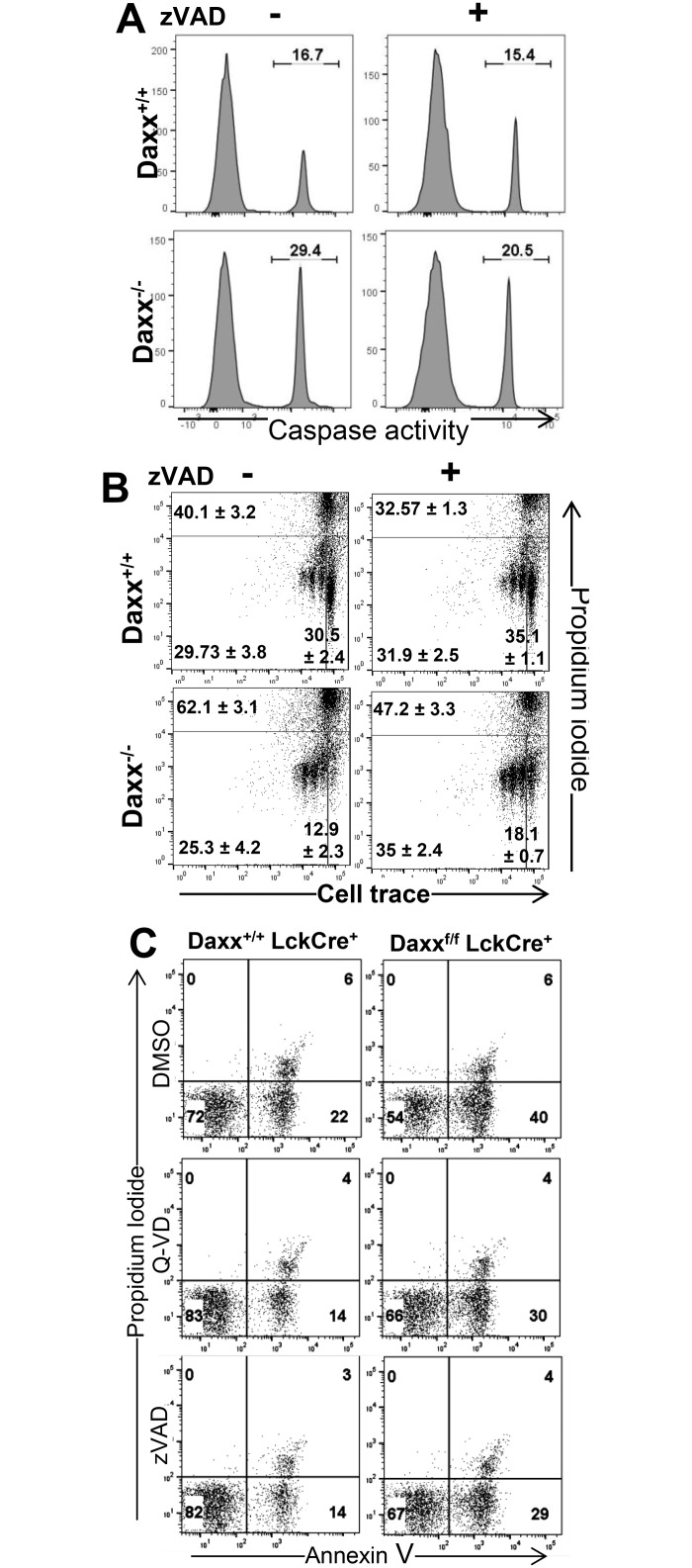
Daxx deletion leads to caspase activation and apoptosis. (A), T cells were incubated with or without zVAD and activated with agonistic anti-CD3/CD28 abs for 12 h and caspase activity was measured using CellEvent Caspase-3/7 Green Detection Reagent. (B), T cells were labeled with CellTrace violet, preteated with or without zVAD, and then activated with agonistic anti-CD3/CD28 abs. At 48 h, T cells were collected and stained with PI and analyzed by two-color flow cytometry. Percentages (±SEM, n = 3) of undivided (CellTrace violet^+^PI^-^) and divided cells (CellTrace violet^lo^PI^-^) and cell death (PI^+^) are indicated. (C), T cells were pretreated for 1 h with the vehicle or indicated caspase inhibitors, and were activated with anti-CD3/CD28 abs for 14 h. T cells were then stained with Annexin V and propidium iodide, and two color flow cytometric analysis was performed.

To understand the mechanism of the enhanced cell death response in *Daxx*^*-/-*^ T cells, caspase activity was measured through CellEvent Caspase-3/7 Green Detection Reagent to determine whether the enhanced cell death is due to apoptosis. This analysis showed that caspase activity was greatly increased in *Daxx*^*-/-*^ T cells compared to wild type T cells after 12 h of TCR activation (left panel, Fig 5A). When T cells were pretreated with the caspase inhibitor z-VAD-fmk, caspase activities in *Daxx*^*-/-*^ T cells was reduced to near wild type levels (Right panel, Fig 5A). To determine whether this caspase activity was responsible for the enhanced cell death response in activated *Daxx*^*-/-*^ T cells, zVAD treatment of *Daxx*^*-/-*^ and *Daxx*^*+/+*^ T cells followed by TCR stimulation was performed. Proliferation and cell death were detected by two-color CellTrace Violet and PI flow cytometry analysis. Similar to the data in Fig 4D, higher percentages of the PI^+^ population (dying/dead cells) as well as divided populations were observed in activated *Daxx*^*-/-*^ T cells than in control *Daxx*^*+/+*^ T cells (right panel, Fig 5B). However, zVAD-treated *Daxx*^*-/-*^ T cells had less cell death compared to untreated *Daxx*^*-/-*^ T cells after 48 h of TCR activation (60.6% vs. 45.1%, respectively), while faster cell division in activated *Daxx*^*-/-*^ T cells was not affected (right panel, Fig 5B). Therefore, caspase activation appears to play some role in enhanced cell death in Daxx-deficient T cells. In further analysis, greatly increased Annexin V^+^PI- cells were present in *Daxx*^*-/-*^ T cells, and caspase inhibitors, zVAD and Q-VD, partially inhibited *Daxx*^*-/-*^ T cells death (Fig 5C).

**Fig 6:**
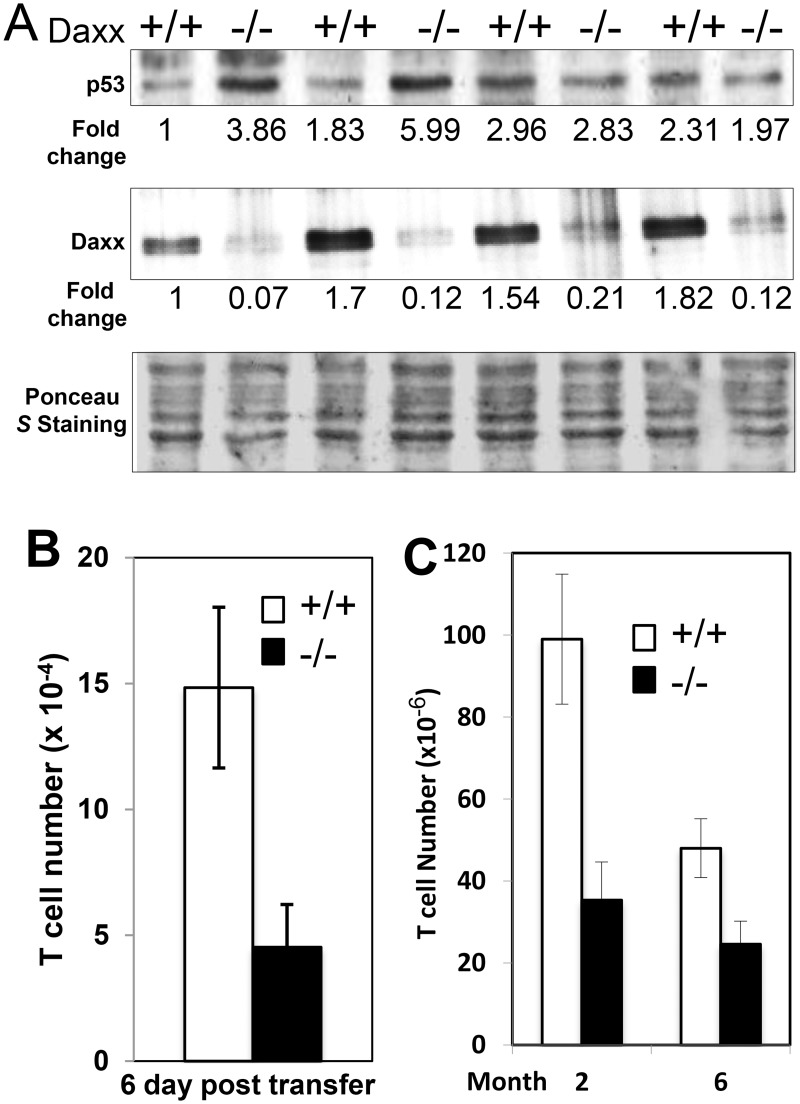
Normal p53 expression and decreased *in vivo* survival in *Daxx*^*-/-*^ T cells. (A), T cells were stimulated through CD3 and CD28 for 24 h, and the levels of the indicated proteins were analyzed by western blotting. Numbers indicate fold changes. Ponceau *S* was used to stain the membrane to visualize proteins transfer. (B.), CD45.2 T cells were adoptively transferred into sublethally irradiated CD45.1 host mice, and T cells in the spleen and lymph nodes were isolated and enumerated 7 days post transfer. (C), T cells numbers in the spleen and lymph nodes of 2-month-old mice were compared with 6-month-old mice (n = 6).

T cells may undergo apoptosis through the p53-mediated pathway. Previous studies had indicated that Daxx plays a role in regulating posttranslational stability of the p53 tumor suppressor through interaction with the Mdm2/Hausp complex [37]. However, no consistent difference in p53 expression was observed between *Daxx*^*-/-*^ and *Daxx*^*+/+*^ T cells (Fig 6A). *In vivo* survival of *Daxx*^*-/-*^ T cells transferred to sublethally irradiated hosts were also greatly diminished compared to *Daxx*^*+/+*^ control T cells (Fig 6B). Significant numbers of T cells were still present in older (6 month) *Daxx*^*t-/-*^ mice (Fig 6C), indicating that the T cell deficiency phenotype in young mice (2 month) does not become proportionally more serious as mice age.

## Discussion

The primary objective of the current study is to address a long-standing enigmatic issue regarding the role of Daxx in Fas-induced apoptosis. To this end, our data showed that conditional deletion of Daxx does not lead to progressive accumulation of T cells in the periphery, a hallmark of the *lpr* disease in *Fas*^*-/-*^ mutant mice. To the contrary, the peripheral T cell pool was decreased upon T cell-specific deletion of Daxx (Figs 1 and 2). Moreover, no defect in Fas-induced apoptosis was detected in *Daxx*^*-/-*^ T cells (Fig 3). Although embryonic development was disrupted when Daxx was deleted in germ cells, as shown in the previous study [29], the thymic T cell population was unaffected when Daxx was deleted after T lineage commitment (Fig 2B). Therefore, while essential in embryonic cells, Daxx is dispensable in thymocyte development. Importantly, the current study reveals a novel function for Daxx in mature T cells survival *ex vivo* (Figs 4C and 4D and 5A and 5B) as well as *in vivo* (Fig 6B and 6C). In total, our data indicate a novel mechanistic insight that Daxx suppresses activation-induced cell death in mature T cells.

Regulation of apoptosis plays a critical role in the immune system. Extrinsic cell death through Fas signaling helps maintain immune system homeostasis and suppress autoimmune disease. The challenge in analysis of Daxx function lies, in part, in the essential role for Daxx in early embryonic development, precluding proper studies without viable *Daxx*^*-/-*^ mice. Prior studies depended largely on the use of a dominant negative Daxx mutant, which may have unintended effects [20–22]. For example, using the same Daxx-DN mutant, two independent studies reached opposing conclusions regarding the function of Daxx in Fas-induced apoptosis [27,28]. To date, the only other available system is a mouse *Daxx*^*-/-*^ embryonic stem cell line, which was used in understanding a Fas-independent function for Daxx in controlling Mdm2 stability [37]. However, a major role for Fas is inducing apoptosis in lymphocytes. The data using a conditional Daxx knockout mouse model demonstrate that Daxx does not play a role in Fas-induced apoptosis in immature T cells, nor in resting or activated mature T cells (Fig 3). By contrast, Daxx deficiency leads to enhanced death in T cells activated through the TCR (Fig 4C–4E).

Although Daxx binds to Fas in yeast cells, the interaction between the endogenous proteins could not readily be detected in mammalian cells [20–22]. In contrast, FADD is readily recruited to Fas in a stimulatory signal-dependent manner and can be detected using conventional co-immunoprecipitation assays [13–15,38]. The DD-containing carboxyl half of FADD functions as a potent dominant negative mutant (FADD-DN), blocking endogenous FADD-Fas interaction and apoptosis induced by stimulation of Fas [13,15]. Interestingly, FADD deficiency blocks embryonic development [39]. Conditional FADD deficiency renders T cells and B cells resistant to Fas-induced apoptosis [32,40]. However, FADD deletion does not lead to the *lpr*-like lymphocyte accumulative-autoimmune diseases [32,40], as seen in Fas-deficient mice. Similarly, transgenic expression of FADD-DN blocks Fas-induced apoptosis without causing *lpr* disease [41–43]. This has raised the possibility that Daxx may direct a parallel, FADD-independent, death signal leading from Fas. However, recent studies demonstrated that absence of FADD unleashes a necrotic death, which is dependent on RIP1 and RIP3 [44,45].

Promiscuous interaction of Daxx with many proteins, depending on various *in vitro* assays used, has been noted previously [29]. Although Daxx-DN could block Fas-induced apoptosis *in vitro* using various non-lymphoid cell lines [20–22,26,46], further validation using primary Daxx-deficient cells has not been carried out until the current study. Our data show that Fas-induced apoptosis proceeds normally in T cells without Daxx (Fig 3). If there is abnormality in cell death responses, Daxx-deficient T cells die more readily when activated (Fig 4C–4E). Indeed, *ex vivo* accumulation of activated Daxx-deficient T cells was significantly reduced compared to wild type control T cells (Fig 4E). This may partially explain the reduced T cell pool in the periphery when Daxx is deleted (Figs 1D and 2). Although Daxx is important in mature T cell survival, it appears to play an insignificant role in immature T cells in the thymus (Fig 2B). Although the data show enhanced caspase activation and increased annexin V^+^PI^-^ population, caspase inhibitors displayed an incomplete effect on death in *Daxx*^*-/-*^ T cells (Fig 5). This indicates that apoptosis only plays a partial role in death responses in *Daxx*^*-/-*^ T cells. Adoptive transfer experiments indicate increased death in *Daxx*^*-/-*^ T cells *in vivo* (Fig 6B). In the current model, Daxx expression is intact in hematopoietic stem/progenitor (HSC/P) cells, and deletion of Daxx is not induced in the T lineage until development proceeds to immature T cells. Therefore, it is unlikely that the conditional *Daxx*^*t-/-*^ mutant mouse would be completely devoid of T cells as they age, given that HSC/P cells are Daxx-sufficient. This is indeed that case, as shown in Fig 6C.

In conclusion, the current study helps clarify that Daxx is dispensable in Fas-induced apoptosis and does not play a role in regulating p53 turnover in T cells. Importantly, the data revealed a novel role for Daxx in maintaining the peripheral mature T cell pool without affecting T cell development in the thymus. Daxx is not only present in cytoplasm but also performs certain functions in the nucleus. Future studies will aim on in-depth understanding of the pathways that Daxx regulates in T cells and additional cell types.
